# Minutes and Discussions before the Eastern Indiana Dental Association

**Published:** 1875-08

**Authors:** 


### MINUTES AND DISCUSSIONS BEFORE THE EASTERN INDIANA DENTAL ASSOCIATION.

The first session of the Eastern Indiana Dental Association convened at the rooms of the Y. M. C. A., and was called to order by the President at 2 p. m., with the following members present: Drs. Jamison, Stanley, Ellis, Shelby, White, Chappell, C. S. Wilson, W. N. Wilson, Bushong and Wagoner.

Drs Stanley, Ellis and Chappell were appointed as a board of censors, and reported favorably upon the following names, who were elected as members of the Association: Drs. J. W.

Jay, of Richmond; Welb Hays, of Rushville; and C. B. Riggs of Manilla.

Drs. J. L. Braffutt, L. B. Taylor and Boyd were elected as honorary members.

The first order of business was called and passed on account of the absence of the Essayist. "Idiosyncrasies of the patient to be considered as to the failure or success in filling teeth" was then taken up, and quite a discussion ensued, participated in by Drs. Chappell, Stanley, White, Braffutt, Jay, Ellis and Jamison, in which these facts, among many others were set forth, that it was very important that the confidence of the patient should, if possible, be secured and their timidity overcome before attempting to operate.

Under the 3d order of business, Dr. Jay presented a paper on Alveolar Abscess and its treatment, offering several cases in practice of much interest, and was followed by Dr. Chappell with an instructive essay upon the "Treatment of dental pulps and pulpless teeth," both essays eliciting interesting discussions.

"Sensitive Dentine and Pain Obtunder" was next considered, in which the latter in a general way was condemned, its application being attended with variable results. Dr. Jay reported having used arsenic in small quantities with good results in sensitive dentine.

*Subject, Mechanical Dentistry.* Dr. Jay remarked that in his vicinity much was done in the way of cheap artificial teeth. They are constantly being palmed off upon the people, as good work, and such work is constantly referred to by the people, as much cheaper than some dentists will work. This is a very serious difficulty with which the competent and honorable dentist has to contend, especially in a country like this. It is true this cheap work rarely if ever gives satisfaction, but then it affords ground for complaint against better work. This state of things compels many to work for lower prices than they otherwise would; and in this way they become ultimately demoralized, some good men in their attempt to compete with cheap teeth and cheap men and worthless work, come to very low prices for the purpose of convincing the people that there is a better way.

Dr. Hays remarked that the people in his section, (Rushville,) are becoming awakened to the necessity of securing competent dentists, having been imposed upon very greatly. He regards rubber as the great cause of this state of things and he hoped to soon return to the good old way of gold plate for artificial teeth.

Dr. Stanley said he knew very little of the status of mechanical dentistry, for some time had but little to do with it—engaged in operating principally.

Dr. Wells, of Indianapolis, said he had done nothing at artificial teeth for months.

Dr. Ellis said he recommended good artificial teeth and wanted the regular price for his services and was not in favor of cheap work nor doing any injustice to his work or family for the sake of competing in low prices and believed he did as much work as those that made the price the scale of competition instead of good service.

Dr. Chappell said mechanical dentistry has been supplying a very important work, and as every thing has its day, and the dentists' mellenium approaches, we soon hope to dispense with all necessity for artificial teeth; but those that have been compelled to use them should join in solid battle array against this evil afflicting the future generations. Every dentist should use his entire professional energy in advising and operating for the preservation of natural teeth, and in this age there is need of a fine dental operator for every township throughout the state, and in same proportion for the towns and cities; and as there is much difference in price of artificial teeth as in operating by different dentists.

Dr. Budd believed that there was more demand for improvement with the dentists in saving the teeth rather than to sacrifice them for the purpose of our gaining more proficiency in making artificial ones, and that mechanical dentistry no doubt was enjoying its own proper sphere, owing to the cheapness of teeth and poor operators on natural teeth and bad mechanics in the mechanical branch, and the want of true cultivation of the people to the importance of saving their natural teeth is the only salvation for the people or the profession, and the only standard by which our proper sphere as a specialty or distinct profession can be maintained.

Dr. Jamison said we can not expect the people to be cultivated in the value of substantial dental practice unless the dentist takes the responsibility to do the right and with dignity maintain it, if cheap teeth on rubber was right he failed to see it, and had for a long time advised against the use of it by respectable dentists and the patients generally, and was glad to know that dentists were discontinuing the business of artificial teeth and rubber especially when they were compelled to make them. So long as some dentists take students, as in some cases, by the half dozen at a time, exacting from them $50 to $200 each, and in a few weeks starting them out as Dr. so and so to pull out teeth and put in artificial substitutes at any price they can get, so long will the people be imposed upon and cheap dentistry be in the land. If members of the societies who subscribed to our code of ethics would live to their promises and not violate the obligations of a gentleman of honor, much could be accomplished in the way of true dental practice and the people be better served, and the profession highly honored.

Dr. Webb Hays, of Russellville, submitted a very extraordinary specimen of teeth. Three teeth, crowns and half of roots, two bicuspids, one molar resting in a piece of bone one and half by two and a half ins. by one eighth inch thick, porous and laminated and corrugated, the molar appeared to be carious, dentine nearly all gone, over half of enamel perfect, bicuspids perfect, the case was one of "extra uterine fœtition" the lady had given birth to two children before the appearance of the tumor and two since the appearance.

### REPORT OF AUTOPSY.

Mrs. M., aged 59 years; body very much emaciated, great enlargement of abdomen and epigastric—scalpel introduced in the enlargement in the middle of left lumbar region, wall thick, knife cutting with a gritty feel—hints of 1st serous fluid and lastly of serous mixed with pus—after fluid passed there was great redundancy of tissue, on opening the cavity it was a thick, corrugated wall with bony deposits over its whole extent with an occasional process of enamel with perfectly formed teeth. The general appearance of the inner wall of the sac was that of coral deposit the sac was adherent to the convolutions of the intestines. Below it was partially free. Fimbrinated extremities were adherent to and lost in the sac, a large tumor formed the upper part of the anterior portion of the wall of the sac about seven inches in length, four and half in breadth, concave on both surfaces, two and one half inches in thickness. On opening longitudinally filled with sero-purulent matter, on removing the fluid, the cavity was found to be divided into fossa, and sinuses with bony walls and septums having the general appearance of brain cavity.

Uterus normal in situ, fallopian tubes pass off to either side properly degenerated over the sac, fimbriated extremities, adherent to and lost in the large abnormal sac at their respective sides.

Bladder adherent to pelvic wall tissue soft, friable, easily broken with finger, filled with purulent matter, kidneys normal in size, weight and color; spleen thickened and softened; appearance on being cut of a mass of clotted blood, liver enlarged, grayish, soft in texture, adherent, lower border on right side to abdominal walls, lungs normal, adherent posteriorly; heart natural in size, sac filled with serum.

